# The Relationships between Economic Scarcity, Concrete Mindset and Risk Behavior: A Study of Nicaraguan Adolescents

**DOI:** 10.3390/ijerph17113845

**Published:** 2020-05-28

**Authors:** Pilar Aguilar, Amparo Caballero, Verónica Sevillano, Itziar Fernández, Dolores Muñoz, Pilar Carrera

**Affiliations:** 1 ETEA-Instituto de Desarrollo, Universidad Loyola Andalucía, 14004 Córdoba, Spain; mpaguilar@uloyola.es; 2 Department of Social Psychology and Methodology, Universidad Autónoma de Madrid, 28049 Madrid, Spain; veronica.sevillano@uam.es (V.S.); lola.munnoz@uam.es (D.M.); pilar.carrera@uam.es (P.C.); 3 Department of Social and Organizational Psychology, Universidad Nacional de Educación a Distancia, UNED, 28040 Madrid, Spain; ifernandez@psi.uned.es

**Keywords:** poverty, construal level, fatalism, risk behaviors

## Abstract

Background: Nicaragua is one of the poorest countries in Latin America, with an extremely low human development index (HDI). Fifty-two percent of the Nicaraguan population are children and adolescents under 18 years of age. Nicaraguan adolescents present several risk behaviors (such as teenage pregnancies, consumption of alcohol, tobacco, cannabis). Our study examines the links between risk behaviors, fatalism, real economic scarcity, and concrete construal level for adolescents with low and middle-low socioeconomic status in Nicaragua. Methods: Nicaraguan adolescents (*N* = 834) from schools located in especially vulnerable areas (low economic status) or in neighborhoods with middle-low social class completed several scales and questions to evaluate fatalism (SFC—social fatalism scale), construal level (BIF) and their past and future risk behaviors (smoking cigarettes, smoking cannabis, unsafe sex, and alcohol consumption). Results: We identified that the poorest individuals who maintained a concrete style of thinking had the highest rates of past and future risk behaviors. This vulnerable group also reported the highest levels of fatalism, i.e., negative attitudes and feelings of helplessness. Encouragingly, the adolescents who were able to maintain an abstract mindset reported healthier past and future habits and lower fatalism, even when they belonged to the lowest social status. In the middle-low economic group, the construal level was not as relevant to maintaining healthy habits, as adolescents reported similar rates of past and future risk behavior at both construal levels. Conclusions: All these results support the importance of considering construal level when studying vulnerable populations and designing risk prevention programs.

## 1. Introduction

Nicaragua, with an estimated population of 6 million inhabitants, is one of the poorest countries in Latin America with the lowest levels of development [1].

Data from the latest human development index (HDI) in 2018 (Middle HDI) indicate that with an HDI of 0.651, Nicaragua ranks number 126 out of 189 countries. Moreover, 6.2% of Nicaragua’s population lives on less than 1.9 dollars a day, and 29.6% lives below the national poverty line [1]. 

Fifty-two percent of the Nicaraguan population is children and adolescents under 18 years of age. Several risk behaviors are present among Nicaraguan adolescents. The number of teenage pregnancies in this country is one of the highest in Latin America [2], with a low prevalence of condom use in this population [3]. Regarding the consumption of substances (e.g., alcohol, tobacco, cannabis, and cocaine), Nicaraguan adolescents are very vulnerable to the use of illegal substances, as their country is a main transit point of drug trafficking [4]. Alcohol is the most commonly used substance in Nicaragua, especially among adolescents [5]. Importantly, the World Health Organization [6] has warned that many serious adult diseases have their origins during adolescence. These figures and arguments support the relevance of studying the factors that promote risk behaviors in Nicaraguan adolescents. Many explanations typically focus on the environmental conditions associated with poverty (e.g., lack of access to healthy food). Although these situational variables clearly contribute to explaining the link between poverty and health, other factors can help us to understand how poverty affects health-risk behaviors; we propose the construal level or way of thinking. Although the construal level can be considered a personal disposition [7], it can also be changed by external procedures, which opens new ways of social intervention. 

Does poverty favor a different way of thinking? When a person mentally represents an action or object, he/she can focus on different aspects, and the features considered will influence his/her decisions. For instance, if individuals consider the action of smoking and focus on its negative future health consequences, they will be motivated to not smoke; however, if individuals think about the short-term relaxing effects of smoking, they will probably decide to smoke. Knowing how different social groups mentally represent relevant actions can help us to explain the differences in their actual behavior, which is especially important in relation to health-risk behaviors among vulnerable poor adolescents.

Action identification theory (AIT) [7,8], pointed out that individuals can mentally represent an action at different levels of abstraction. A low-level representation indicates how an action is performed (concrete mindset) and involves viewing the action in terms of its details or mechanics, while a high-level representation focuses on why the action is performed (abstract mindset) and is concerned with the consequences and implications of the actions. Later, construal level theory (CLT), Trope and Liberman referred to not only actions, but also objects and events [9,10]. Trope and Liberman proposed that people consider different features to construe a mental representation of an event depending on their styles of thinking [9]. They bidirectionally connected construal level with psychological distance (temporal, spatial, social, and hypothetical). According to CLT, a higher psychological distance involves higher-level mental representations, and correspondingly, a more abstract representation of an event involves placing the event at a greater psychological distance. An individual with an abstract mindset represents events by focusing on the central, abstract characteristics and the final outcomes, rather than on the peripheral, concrete subordinate traits and the immediate consequences, which are considered under a concrete mindset [9,11]. Thus, when a person thinks about a specific behavior (e.g., snacking) in an abstract way, he/she focuses on the distant outcomes of the action and thinks about why he/she is engaging in the behavior (e.g., to maintain energy and health). However, when an individual has a concrete style of thinking, he/she focuses on the immediate consequences of the action and thinks about how to do the behavior (e.g., to eat something that is tasty and easily available). Different representations of the same action promote very different behaviors.

Both types of construal (abstract vs. concrete) have important consequences for behavioral decisions, especially when the behaviors considered are related to health and wellbeing. Most health and prevention actions lead to desirable outcomes in the long term, but undesirable consequences in the short term; this imbalance is associated with several self-control problems. Self-control refers to the acceptance of immediate obstacles and inconveniences in the present to obtain benefits in the future. The lack of attention to immediate benefits requires important cognitive and affective efforts that individuals with an abstract style of thinking seem to carry out more easily. Accordingly, an abstract mindset promotes a stronger focus on final goals than on present means, facilitating self-control [9,11,12,13,14,15,16,17]. Following our example about snacking, a person thinking abstractly will probably choose a healthy snack (e.g., a piece of fruit), while a person thinking concretely will probably choose the most readily available and delicious option (e.g., a chocolate bar). Research on self-control has supported these predictions [16].

Theoretical models focused on a temporal perspective have supported similar predictions [18]. Individuals with a future orientation “consider the potential distant outcomes of their current behaviors” ([18]; p. 743); they report more healthy behaviors than those without a future orientation. Future orientation has been associated with more sport practicing [19,20], more condom use [21,22], lower levels of tobacco and alcohol consumption [18], less aggressive behavior [23], and a healthier diet [24,25].

Although the style of thinking can be considered a personal disposition [7], it can also be modified by external cues. AIT suggests the difficulty of an action as the main factor that influences the level of abstraction, while CLT proposes psychological distance as the main factor. Thus, when an individual perceives the difficulty of an action or is overwhelmed by the urgency of a situation, a concrete or low-level style of thinking emerges, making the means to perform the action more important than the final goal. However, when an action is perceived to be easy and effortless or the person takes a long-term perspective, the final goal becomes more relevant.

Contextual cues, such as the perception of difficulty associated with actions (i.e., obstacles), or the urgency of a situation (i.e., short-term frame) favor a concrete style associated with lower self-control and worse health and wellbeing. The difficulty and urgency of an action depends not only on the action itself, but also on personal (mental, physical, and environmental) resources. When people suffer from poverty, they face numerous obstacles in their daily lives. People in need have to attend to immediately available options and make quick decisions without the time or means to look for better alternatives. Imagine a person with serious financial concerns who must decide between paying for a medical test to prevent a future severe illness or using that money for urgent bills. What will this person decide? Urgent decisions and demanding situations promote a short-term perspective that diminishes the importance of future consequences. Research on other kinds of threats has found that individuals who feel mortally threatened pay more attention to immediate rewards (presentism); this effect is even stronger for participants who grew up in resource-scarce contexts [26].

Previous research has shown that individuals experiencing scarcity are more likely to show a cognitive bias referred to as “tunnel-vision thought”, associated with decisions that involve undesirable long-term consequences [27,28]. In both simulated [28] and real shortage situations [27], people shifted their attention toward short-term outcomes, even though those decisions involved disastrous long-term results. Thus, in experimental games, “poor” participants overborrowed and neglected useful information about the future more than “rich” participants [28]. These results are coherent with real-world data, showing that people with low income borrow too much, a decision that reduces their chances to escape poverty [29]. Hansen, Kutzner and Wänke [30] showed that after the activation of the concept of money, participants considered high-level and central features of products (e.g., the quality of sound of a radio) to be more important than secondary characteristics (e.g., extra accessories, such as a clock on the radio). Considering all these previous results, it becomes clear that scarcity is a threat and a disadvantage; a low-income situation not only presents fewer resources and more difficulties, but also prevents people from paying attention to future long-term consequences of their actions. Being blind to the future makes it more difficult to break the circle of poverty.

The picture can be even worse; a lack of attention to future consequences by poorer people might be related to a perception of the world as unpredictable. People experiencing scarcity may feel less control over their situations, because they do not usually make future predictions, but without thinking about the future, they cannot anticipate the difficulties to better solve them. Manstead [31] found that social classes with fewer economic resources are more likely to explain their current life situations based on external factors, and to show lower levels of perceived control. Similarly, poverty is associated with fatalism, especially in Latin America [32,33]. Social fatalism has been defined as the perception of having no power to influence one’s own actions and as passive feelings of resignation about the future (e.g., I can do nothing to change our situation; [34]). Supporting the negative consequences of fatalism, previous research has linked fatalism with a variety of health problems and risk behaviors, such as diabetes [35,36], cardiovascular diseases [37], and unsafe sex practices [38]. Poverty makes individuals focus on their daily present difficulties, i.e., presentism [39], and ignore the uncertain future [40]. From a construal-level perspective, people in situations of scarcity are more focused on their day-to-day survival than on planning for a future that they feel they cannot control (lack of control/predeterminism); these features define a concrete mode of thinking.

## 2. Present Research

This study examines the links between real economic scarcity, concrete construal level, fatalism and risk behaviors (i.e., smoking cigarettes, smoking cannabis, unsafe sex, and alcohol consumption) for adolescents with low and middle-low socioeconomic status in Nicaragua.

In the present research, we expect that adolescents suffering from the highest level of economic scarcity will present more concrete styles of thinking than adolescents of middle-low economic status, and that this difference will explain the higher rates of past and future risk behaviors and greater fatalism among the former group of adolescents. In addition, we expect that individuals with higher income (i.e., middle-low status) will show more abstract mindsets associated with fewer risk behaviors and lower levels of fatalism. Considering the positive effects of abstraction, we also expect that, among poorer individuals, those who are able to maintain an abstract style of thinking will report less frequent past and future risk behaviors and lower levels of fatalism.

## 3. Method

### Participants and Procedure

The sample comprised 834 participants between 12 and 16 years old (489 females; *M*_age_ = 13.35; *SD* = 1.22) from nine schools, five of which are located in especially vulnerable areas (463 participants, 258 females), characterized by low economic status, while the other four are located in neighborhoods with middle-low social class (371 participants, 231 females). This economic classification was made following the estimations and figures included in the educational projects conducted by each school. When the educational project detailed that the families were suffering severe unemployment, child labor (minor farm works) and problems in basic services as school public transport, the school was classified in the low economic status condition. When the schools reported a better employment situation in the families (i.e., parents worked in commerce, services, factories, agriculture or livestock), children did not have to do minor works for family support, and public services worked properly; the schools were categorized in the middle-low social condition. This classification was supported by professionals working in the Movement for Integral Popular Education and Social Promotion (Fe y Alegría). Fe y Alegría’s workers had an extensive experience with families and schools and their categorization matched with our division into two groups of low and middle-low status.

School teachers administered the questionnaires during regular classes, the questions and scales used in the present research were answered in a single session (approximately during 45 min) and teachers solved questions when they were asked by students. Families and teachers agreed to participate. The survey was presented as a strategy to collect information to better know the situation of Nicaraguan adolescents. Students were guaranteed anonymity and confidentiality. Participation was voluntary and all students who were attending the class during the survey agreed to participate. The questionnaire was integrated in a larger survey that included other measures unrelated to the hypotheses raised in the present research. All schools and families approved the procedure; fulfilling all ethical standards required in psychological research.

**Ethical approval:** All procedures performed in studies involving human participants were in accordance with the ethical standards of the institutional and/or national research committee and with the 1964 Helsinki declaration and its later amendments or comparable ethical standards. 

## 4. Measures

Participants completed several scales and questions, which were presented in the following order:

Fatalism. The social fatalism scale (SFC) [41] was used to measure fatalism. This scale consists of 17 items with a response format scored from 1 (strongly disagree) to 6 (strongly agree). The scale includes four dimensions: predetermination (six items, α = 0.78) (e.g., “I think there is a pre-written script about things that are going to happen in life”); lack of control (four items, α = 0.637) (e.g., “I do not have the ability to change things”); pessimism (three items, α = 0.641) (e.g., “One cannot trust in people”) and presentism (four items, α = 0.645) (e.g., “All that matters is the present, the ‘here’ and ‘now’”). The global fatalism score is computed by averaging all the items (α = 0.83).

Dispositional construal level. Construal level was measured with a short version of the behavioral identification form (BIF) [7,11]. Participants are presented with 14 actions and are asked to choose between two options for each action. One option describes the action in concrete terms (low level), whereas the other option describes the action in abstract terms (high level). For example, participants must choose whether “locking a door” is best described as “securing the house” (high level or abstract level; scored as 1) or “putting the key in the lock” (low level or concrete level; scored as 0). The number of high-level descriptions serves as a measure of abstraction: higher scores indicate higher abstraction. Cronbach’s alpha was acceptable (α = 0.69).

Risk behaviors. Participants rated the frequency of their past behavior during the last six months and their future behavioral intentions for the following risk behaviors: tobacco smoking, alcohol consumption, cannabis use and unsafe sex. The response format was a 7-point Likert scale ranging from 1 (not at all) to 7 (very much). Behaviors were discussed with Fe y Alegria’s workers and school teachers. They considered these behaviors as the most frequent risk actions among Nicaraguan adolescents. Finally, participants reported sociodemographic data about their age and gender.

## 5. Results

### 5.1. Relationships between Economic Status, Construal Level, and Fatalism

First, we calculated the correlations between construal level (abstraction), the four fatalism subscale scores, the fatalism global score (average of all the subscale scores) and the socioeconomic status dummy coded (as 0 = low status, 1 = middle-low status) for the total sample (see Table 1).

The results showed a negative and significant association between an abstract style of thinking and the fatalism scores, showing that when adolescents were focused on distant future (abstract mindset), they reported lower fatalism. 

Construal level did not show a significant relationship with pessimism or presentism (subscales of fatalism). As expected, the correlations between the four fatalism subscale scores and the global fatalism score were positive and significant.

The relationship between economic status and construal level was positive and significant, while the relationships between economic status, the global fatalism score and the fatalism subscale scores were negative and significant in all cases.

To examine these relationships in more depth, we conducted ANOVA tests to explore the influence of economic level (low vs. moderate-low) on construal level and fatalism. Table 2 shows that participants in the lowest economic class had the highest scores for fatalism (and all subscales) and the lowest level of abstraction (i.e., a concrete mindset).

### 5.2. The Influence of Construal Level and Economic Status on Risk Behaviors and Fatalism

First, we conducted a series of ANOVA tests to explore the influence of economic status (low vs. middle-low) on past risk behaviors (tobacco smoking, cannabis use, unsafe sex, and alcohol consumption) and on future behavioral intention. The influence on each risk behavior was examined separately, and the risk as a whole was considered using multi-risk indices. These composite indices were calculated by adding the scores of the four risk behaviors considered for past behaviors (*α* = 0.74) and future intentions (α = 0.79). Table 3 shows the results.

These results showed that adolescents in the lowest economic class reported the highest rates of past risk behavior and future behavioral intentions for each behavior (*p*’s < 0.01) and the multi-risk behavioral indices (*p*’s < 0.001). Even for those behaviors with low frequencies, the gap between the two economic levels was significant.

Secondly, we tested the influence of each mindset (concrete vs. abstract) on each risk behavior (past and future), separately and on the risk as a whole (i.e., the multi-risk indices). We split the sample into two groups using the first and fourth quartiles of the BIF scores. The first quartile was considered the concrete group (*N*_conc_ = 278), with participants with BIF scores ≤8, and the fourth quartile was considered the abstract group (*N*_abst_ = 230), with participants with BIF scores ≥12. The abstract group presented the lowest level of past and future risk behavior (see Table 4).

After calculating post-hoc comparisons between groups (DMS tests), we explored the combined influence of economic status and construal level (the first and fourth quartiles of the BIF scores), constructing a categorical variable with four levels: (1) concrete mindset and low status, *N* = 205; (2) concrete mindset and middle-low status, *N* = 73; (3) abstract mindset and low status, *N* = 73; and (4) abstract mindset and middle-low status, *N* = 157. We conducted planned comparisons (comparing the low status-concrete condition vs. the other conditions as a whole) for past behaviors and future intentions. We repeated this analysis using the multi-risk indices. The results are shown in Table 5.

As predicted, adolescents in the lowest economic class with a concrete mindset had the highest rates of past risk behaviors (except for alcohol consumption, for which the differences were not significant) and the greatest intentions to repeat risk behaviors in the future. Results are the same for the multi-risk indices: in the poorest group, adolescents who were able to maintain an abstract mindset reported similar past risk behavior and future intentions as the adolescents in the middle-low class.

When we repeated the planned comparisons using the global fatalism scores (average of all the subscales), the data showed the same pattern: adolescents who were in the lowest class and presented a concrete mindset reported the highest levels of fatalism (see Table 6). The differences between groups (DMS tests) showed that concrete construal level was associated with the highest level of fatalism in both economic groups. An abstract mindset seemed to reduce the differences in fatalism between the two economic groups.

## 6. Discussion

When economically disadvantaged people face daily life decisions, they are prone to make poor decisions that make solving their vulnerable situations more difficult. This tendency is especially important in adolescence, a vital period in which many serious adult diseases have their origin.

Poverty is an endemic trait in Nicaragua, but even in this context of scarcity, it is possible to compare people with extremely low incomes (i.e., low status) with people with slightly better economic conditions (i.e., middle-low status). Our results reveal that economic scarcity was associated with a low construal level and fatalism in Nicaraguan adolescents. The poorest teenagers presented the most concrete style of thinking and the highest fatalism, in terms of lack of control, predetermination, presentism and pessimism. The relationships between construal level and the subscales of fatalism were significant for lack of control and predetermination and almost significant for the other two subscales. All these results support that the most severe poverty is clearly associated with a low-level style of thinking (concreteness) and negative attitudes (fatalism). The obstacles arising from low income seem to lead people to focus on immediate goals and to therefore underestimate future outcomes that will be more beneficial for them most of the time. The poorest individuals are not only placed under pressure by their demanding contexts, but also feel that they cannot face difficulties successfully. This dangerous combination favors passive and indulgent attitudes that consolidate a situation of risk and vulnerability that makes change difficult.

When people experience scarcity, they focus on the present and immediate rewards (e.g., relaxation, social status, and pleasure) which are offered by many risk behaviors (e.g., alcohol, tobacco, and cannabis use). Previous research on the consumption of high-calorie food had showed that poverty and inequality increased calorie intake [42]. Recently, Rogen, Verkooijen and de Vet found a tendency bordering on significance showing that those in the scarcity experimental condition consumed more calories under low hunger [43]. All these results support that when people experience poverty, the immediate rewards become a priority. Our results fit all these previous findings: the poorest adolescents reported the highest rates of risk behaviors in the past six months and the greater intentions to repeat these unhealthy behaviors in the future.

The multi-risk indices better showed the vulnerability of adolescents than the analyses of individual behaviors, because the higher scores indicated that the participants did not practice isolated risk behaviors but several risk behaviors. The results revealed that the poorest participants had the highest scores for multi-risk behavior in terms of past behaviors and future intentions. Clearly, adolescents with lower incomes engaged in more unhealthy habits, as shown in the analyses of each of the risk behaviors studied. Young people with the lowest incomes consumed more cannabis and tobacco, drank more alcohol and practiced more unsafe sex, and unfortunately, these same results were found for future behavioral intentions.

Regarding the role played by construal level, the results supported that the combined influence of concreteness and poverty promoted higher rates of past risk behavior and future risk behavioral intentions. The statistical interaction between construal level and economic status was not significant; however, it was not necessary to support our hypothesis (the 1 vs. 3 pattern can be found without interaction). Indeed, a significant interaction between construal level and economic status may have been found, but it would not have been sufficient to support our hypothesis, because any interaction effects would not have been in line with our hypothesis. We did not expect that people with low status and an abstract mindset would present extremely low risk behavior, which is a result that would have favored the interaction. The expected influence of concreteness and scarcity was focused on the comparison between the low status-concrete group and the other three groups as a whole. The data supported the predicted pattern, showing that adolescents with both the lowest income level and a concrete style of thinking presented the worst pattern of past and future behavior. Only alcohol consumption did not have significant results in the planned comparisons, but the comparisons did show the expected tendency in terms of past behavior.

We can interpret these results in a different and promising way. Abstraction seems to lead people to better resist temptations, which is especially relevant for the poorest adolescents, who are in the most vulnerable situation. Young people, who had an abstract construal level, even when they were in the lowest social class, seemed to be more capable of controlling their behavior and choosing the healthiest options. In the middle-low economic group, the construal level was not as relevant to maintaining healthy habits, because adolescents reported similar rates of past and future risk behavior at both construal levels. A better family economic situation might have facilitated alternative protective strategies, such as greater parental control or healthier leisure options that countered the effect of a concrete mode of thinking in this middle-low economic group.

The results for fatalism also supported the importance of considering construal level when studying vulnerable populations. Adolescents in the worst economic situation who also presented a concrete mindset reported the highest level of fatalism. In contrast, an abstract style of thinking reduced the gap between the two economic groups in terms of fatalism: individuals with an abstract mindset in the poorest group reported lower levels of fatalism than individuals with a concrete mindset in this group; furthermore, the low status-abstract subgroup reported a level of fatalism similar to that of the middle status-abstract subgroup. If young people feel that they cannot do anything to improve their wellbeing, psychosocial interventions may be unsuccessful in changing unhealthy behaviors and harmful attitudes; when this happens in the most disadvantaged group, in which individuals are focused on the immediate, demanding situation, the “perfect storm” emerges.

## 7. Conclusions

Poor people with a concrete mindset focus on immediate rewards and underestimate future damages and costs, making the improvement of their situations more difficult. Encouragingly, adolescents in the lowest class who are able to think in an abstract way seem to make better decisions and to trust more in their ability to control their futures.

The possibility of protecting disadvantaged adolescents through psychosocial interventions aimed at modifying their construal levels, which can be manipulated [11,44,45], remains an open question. Previous research has shown that it is possible to change construal level in natural settings by using simple interventions, such as printing messages on door hangers describing why people should perform an action [46]. In the laboratory, strategies based on construal level primes to promote healthier behaviors have been successful [13] for reducing cigarette consumption. Encouraging adolescents to think about why to perform an action and focus on the long-term future consequences seems to be a promising strategy to improve their wellbeing, even when their economic conditions are very negative. All these results support the importance of considering construal level when studying vulnerable populations and designing risk prevention programs.

## Figures and Tables

**Table 1:** Correlations between abstraction, economic status, fatalism score, and fatalism subscales.

Measures	Abstraction	Fatalism	Lack of Control	Predetermin	Presentism	Pessimism
Abstraction	-					
Fatalism (total)	−0.15 **	-				
Lack of control	−0.16 **	0.74 **	-			
Predetermination	−0.14 **	0.83 **	0.43 **	-		
Presentism	−0.05	0.63 **	0.31 **	0.38 **	-	
Pessimism	−0.02	0.59 **	0.41 **	0.23 **	0.27 **	-
Economic status	0.30 **	−0.16 **	−0.15 **	−0.11 **	−0.09 **	−0.09 **

** *p* < 0.01.

**Table 2:** Means for Low and Middle-low Status adolescents in Construal Level, Fatalism Score, and Fatalism’s Subscales.

Measures	*M*_low_ (SD)	*M*_middle-low_ (SD)	*F* (1, 832)	*ƞ* ^2^
Fatalism	3.34 (0.81)	2.98 (0.84)	20.35 ***	0.024
Lack of control	2.97 (1.15)	2.62 (1.13)	19.83 ***	0.023
Predetermination	3.56 (1.19)	3.29 (1.23)	9.70 **	0.012
Presentism	3.93 (1.21)	3.71 (1.16)	7.14 **	0.009
Pessimism	3.29 (1.22)	3.06 (1.30)	6.75 **	0.008
Abstraction	8.89 (2.59)	10.55 (2.63)	83.63 ***	0.091

** *p* < 0.01; *** *p* < 0.001.

**Table 3:** Frequency and Intention of Risk Behaviors for Low and Middle-low Status Adolescents.

Behavior	*M*_low_ (SD)	*M*_middle-low_ (SD)	*F* (1, 832)	*ƞ* ^2^
Past behavior in the last six months				
Cannabis	1.33 (1.08)	1.07 (0.45)	18.48 ***	0.022
Unsafe sex	1.31 (1.08)	1.05 (0.34)	20.28 ***	0.024
Alcohol	1.58 (1.36)	1.32 (0.94)	9.57 **	0.011
Tobacco	1.32 (1.01)	1.13 (0.55)	10.61 **	0.013
Multi-risk past behavior index	5.55 (3.45)	4.58 (1.67)	24.50 ***	0.029
Intention of behavior				
Cannabis	1.36 (1.18)	1.10 (0.54)	15.40 ***	0.018
Unsafe sex	1.58 (1.42)	1.14 (0.69)	29.60 ***	0.034
Alcohol	1.50 (1.23)	1.33 (0.93)	5.11 *	0.006
Tobacco	1.34 (1.09)	1.12 (0.60)	12.16 ***	0.014
Multi-risk behavioral intention index	5.79 (3.93)	4.69 (2.03)	23.55 ***	0.028

* *p* < 0.05; ** *p* < 0.01; *** *p* < 0.001.

**Table 4:** Contrast between the concrete and abstract mindset in risk behaviors and intention of risk behaviors.

Behavior	*M*_concrete_ (SD)	*M*_abstract_ (SD)	*F* (1, 506)	*ƞ* ^2^
Past behavior in the last six months				
Cannabis	1.34 (1.09)	1.05 (0.29)	14.33 ***	0.028
Unsafe Sex	1.27 (0.98)	1.04 (0.30)	11.71 ***	0.023
Alcohol	1.56 (1.35)	1.35 (0.98)	3.95 *	0.008
Tobacco	1.37 (1.12)	1.12 (0.53)	9.85 **	0.019
Multi-risk past behavior index	5.55 (3.49)	4.57 (1.65)	15.24 ***	0.29
Intention of behavior				
Cannabis	1.32 (1.04)	1.10 (0.64)	7.60 **	0.015
Unsafe sex	1.50 (1.29)	1.19 (0.83)	9.46 **	0.018
Alcohol	1.43 (1.12)	1.29 (0.86)	2.17	0.004
Tobacco	1.30 (0.97)	1.10 (0.66)	6.60 **	0.013
Multi-risk behavioral intention index	5.54 (3.48)	4.69 (2.07)	10.53 ***	0.020

* *p* < 0.05; ** *p* < 0.01; *** *p* < 0.001.

**Table 5:** Post-hoc comparisons between the four groups combining status and mindset in risk behaviors and intention of risk behaviors.

Behavior	*M*_low-concrete_ (SD)	*M*_mod-concrete_ (SD)	*M*_low-abstract_ (SD)	*M*_mod-abstract_ (SD)	Planned Contrast 1 vs. 3 *t* (504)
Past behavior in the last six months					
Cannabis	1.39 (1.17) _a_	1.19 (0.81) _ab_	1.10 (0.39) _b_	1.03 (0.24) _b_	−3.61 ***
Unsafe sex	1.32 (1.08) _a_	1.13 (0.63) _ab_	1.12 (0.59) _ab_	1.00 (0.00) _b_	−3.29 ***
Alcohol	1.60 (1.42) _a_	1.45 (1.14) _ab_	1.46 (1.18) _ab_	1.29 (0.88) _b_	−1.79
Tobacco	1.40 (1.16) _a_	1.30 (0.99) _ab_	1.17 (0.77) _ab_	1.09 (0.37) _b_	−2.48 *
Multi-risk behavior index	5.71 (3.74) _a_	5.08 (2.60) _ab_	4.87 (2.27) _b_	4.42 (1.24) _b_	−3.52 ***
Intention of behavior					
Cannabis	1.38 (1.17) _a_	1.12 (0.47) _b_	1.12 (0.72) _b_	1.09 (0.60) _b_	−3.35 ***
Unsafe sex	1.58 (1.36) _a_	1.29 (1.07) _bc_	1.42 (1.22) _ab_	1.09 (0.53) _c_	−3.01 **
Alcohol	1.41 (1.11) _a_	1.47 (1.14) _a_	1.44 (1.03) _a_	1.23 (0.76) _a_	−0.29
Tobacco	1.33 (1.06) _a_	1.22 (0.67) _ab_	1.12 (0.62) _ab_	1.09 (0.68) _b_	−2.29 *
Multi-risk behavioral intention index	5.69 (3.74) _a_	5.10 (2.59) _ab_	5.11 (2.44) _ab_	4.50 (1.85) _b_	−2.91 **

Means in the same row that do not share the same subscripts differ at *p* < 0.05 in the contrast analysis. In past, behavior differences between low-concrete and mid-concrete were almost significant (*p* ≤ 0.09) in cannabis, unsafe sex and multi-risk past behavior index. * *p* < 0.05; ** *p* < 0.01; *** *p* < 0.001.

**Table 6:** Contrast between the low and middle-low status in fatalism scores.

Groups	*M*_Fatalism_ (SD)	1 vs. 3 Contrast *t* (504)
Low-concrete	3.30 (0.79) _a_	−3.70 ***
Mid-concrete	3.14 (0.74) _ac_	
Low-abstract	3.06 (0.84) _bc_	
Mid-abstract	2.88 (0.81) _b_	

Means in the same column that do not share the same subscripts differ at *p* < 0.05 in the contrast analysis. *** *p* < 0.001.
